# β-III spectrin underpins ankyrin R function in Purkinje cell dendritic trees: protein complex critical for sodium channel activity is impaired by SCA5-associated mutations

**DOI:** 10.1093/hmg/ddu103

**Published:** 2014-03-06

**Authors:** Yvonne L. Clarkson, Emma M. Perkins, Callum J. Cairncross, Alastair R. Lyndon, Paul A. Skehel, Mandy Jackson

**Affiliations:** 1 The Centre for Integrative Physiology and; 2 Euan MacDonald Centre for Motor Neuron Disease Research, The University of Edinburgh, Hugh Robson Building, George Square, EdinburghEH8 9XD, UK and; 3 School of Life Sciences, Heriot-Watt University, John Muir Building, Riccarton, EdinburghEH14 4AS, UK

## Abstract

Beta III spectrin is present throughout the elaborate dendritic tree of cerebellar Purkinje cells and is required for normal neuronal morphology and cell survival. Spinocerebellar ataxia type 5 (SCA5) and spectrin associated autosomal recessive cerebellar ataxia type 1 are human neurodegenerative diseases involving progressive gait ataxia and cerebellar atrophy. Both disorders appear to result from loss of β-III spectrin function. Further elucidation of β-III spectrin function is therefore needed to understand disease mechanisms and identify potential therapeutic options. Here, we report that β-III spectrin is essential for the recruitment and maintenance of ankyrin R at the plasma membrane of Purkinje cell dendrites. Two SCA5-associated mutations of β-III spectrin both reduce ankyrin R levels at the cell membrane. Moreover, a wild-type β-III spectrin/ankyrin-R complex increases sodium channel levels and activity in cell culture, whereas mutant β-III spectrin complexes fail to enhance sodium currents. This suggests impaired ability to form stable complexes between the adaptor protein ankyrin R and its interacting partners in the Purkinje cell dendritic tree is a key mechanism by which mutant forms of β-III spectrin cause ataxia, initially by Purkinje cell dysfunction and exacerbated by subsequent cell death.

## INTRODUCTION

Spectrins are a critical component of the cell membrane skeleton, maintaining cell shape by conferring strength and elasticity (1,2). They associate with the plasma membrane through protein–protein and protein–lipid interactions. Ankyrin is a key component in this network as it binds both to spectrin and transmembrane proteins thus linking spectrin to the plasma membrane (3–9). The importance of ankyrin in maintaining membrane structural integrity is highlighted in erythrocytes where the majority of human hereditary spherocytosis cases actually result from mutations of ankyrin R and not spectrin, even though the common cellular defect is a defective spectrin lattice (10,11).

Within the nervous system, several studies have documented the role of β-IV spectrin and ankyrin G in clustering ion channels along the axon of various neuronal cell types (12–14); however, less is known about the membrane skeleton within dendrites. The β-III spectrin isoform is distributed throughout the soma and elaborate dendritic tree of cerebellar Purkinje cells. Loss of β-III spectrin in mice results in abnormal Purkinje cell dendritic morphology and eventual cell death (15,16). Furthermore, in humans, mutations in β-III spectrin are known to underlie spinocerebellar ataxia type 5 (SCA5) (17) and spectrin associated autosomal recessive cerebellar ataxia type 1 (SPARCA1) (18), both neurodegenerative diseases characterized by gait ataxia and progressive cerebellar atrophy.

Normoblastosis (*nb/nb*) mice are deficient in erythroid ankyrin and have severe haemolytic anaemia (19). In addition, they also develop an abnormal gait and tremor by the age of 6 months, accompanied by a 50% loss of Purkinje cells (20). Ankyrin R is found throughout the cerebellar molecular layer (20,21). Therefore, both β-III spectrin and ankyrin R appear to be essential for Purkinje cell survival with a primary genetic loss of either protein resulting in delayed neurodegeneration and motor deficits. Here, we investigate the interplay between β-III spectrin and ankyrin R and the relevance of their interaction with respect to Purkinje cell dysfunction and cerebellar ataxia.

## RESULTS

### β-III spectrin can recruit ankyrin R to plasma membrane

**Figure 1.**
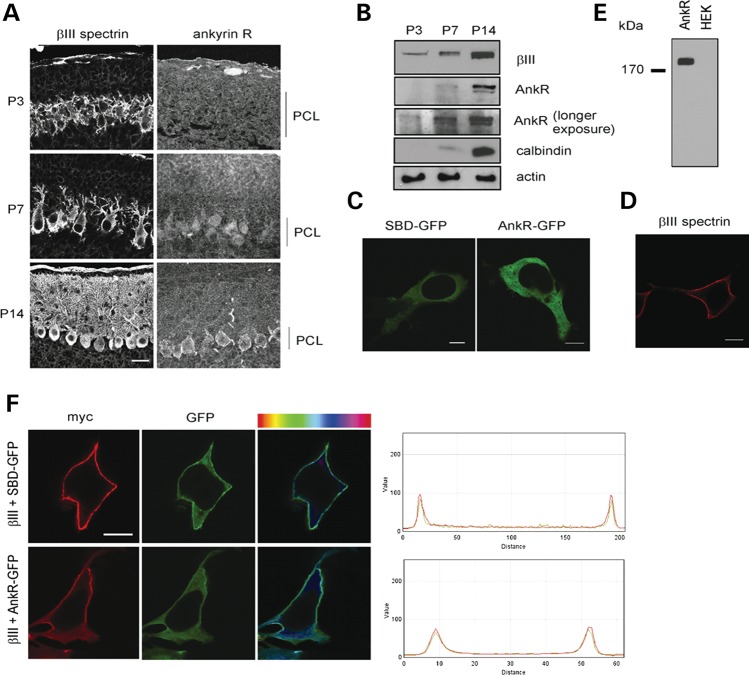
Ankyrin R recruited to membrane by β-III spectrin. (**A**) Sagittal cerebellar sections independently immunostained for β-III spectrin or ankyrin R at P3, 7 and 14. (**B**) Immunoblot analysis of cerebellar homogenates from P3, 7 and 14 animals. (**C**) Representative confocal images of HEK 293T cells transfected with SBD-GFP or AnkR-GFP only. (**D**) Representative confocal image of cell transfected with myc-tagged β-III spectrin only and immunostained using anti-c-myc antibody. (**E**) Immunoblot analysis of untransfected HEK 293T cells and ankyrin R transfected cell homogenates probed with anti-AnkR antibody. (**F**) Cells cotransfected with myc-tagged β-III spectrin and either SBD-GFP or AnkR-GFP. Cells immunostained using anti-c-myc antibody (red). Degree of colocalization shown both by residual map (right column), with cyan representing highest, and histogram of red and green fluorescence intensity through the cell. All images are representative of at least three independent experiments [PCL, Purkinje cell layer; scale bar, 50 μm (A), 10 μm (C, D, F)].

The order in which cytoskeletal components are recruited to the plasma membrane is unclear. Some studies indicate ankyrin recruits spectrin (7), while others suggest conversely that spectrin recruits ankyrin (22). To address this issue, we examined the cellular distribution of β-III spectrin and ankyrin R in Purkinje cells at postnatal days (P) 3, 7 and 14 (Fig. 1A and B). β-III spectrin was already present at the cell membrane at P3, whereas somatic membrane localization of ankyrin R was not evident until P14, although protein was detected by immunoblot at P3 and P7 (Fig. 1B). Therefore, β-III spectrin's localization at the membrane appears to be independent of ankyrin R. To determine whether β-III spectrin can recruit ankyrin R to the plasma membrane, we investigated the interplay between the two proteins in HEK 293T cells (Fig. 1C–F). GFP fusions of full-length ankyrin R (AnkR-GFP) and a truncation containing only the spectrin binding domain (SBD-GFP) of ankyrin R are both predominantly cytoplasmic when expressed individually (Fig. 1C). In contrast, β-III spectrin is found exclusively at the plasma membrane in HEK 293T cells when expressed alone (Fig. 1D). Immunoblot analysis confirmed the absence of endogenous ankyrin R in HEK 293T cells (Fig. 1E). Co-expression with β-III spectrin recruited both forms of ankyrin R to the plasma membrane [Fig. 1F, Pearson's correlation coefficient (R ± SEM) for colocalization: SBD-GFP + β-III spectrin 0.89 ± 0.02; AnkR-GFP + β-III spectrin 0.88 ± 0.07; GFP + β-III spectrin 0.55 ± 0.14; *P* < 0.001, *n* = 15–25]. Therefore, an additional normal function of β-III spectrin is to recruit ankyrin R to the plasma membrane.

### Maintenance of dendritic ankyrin R localization dependent on β-III spectrin

**Figure 2.**
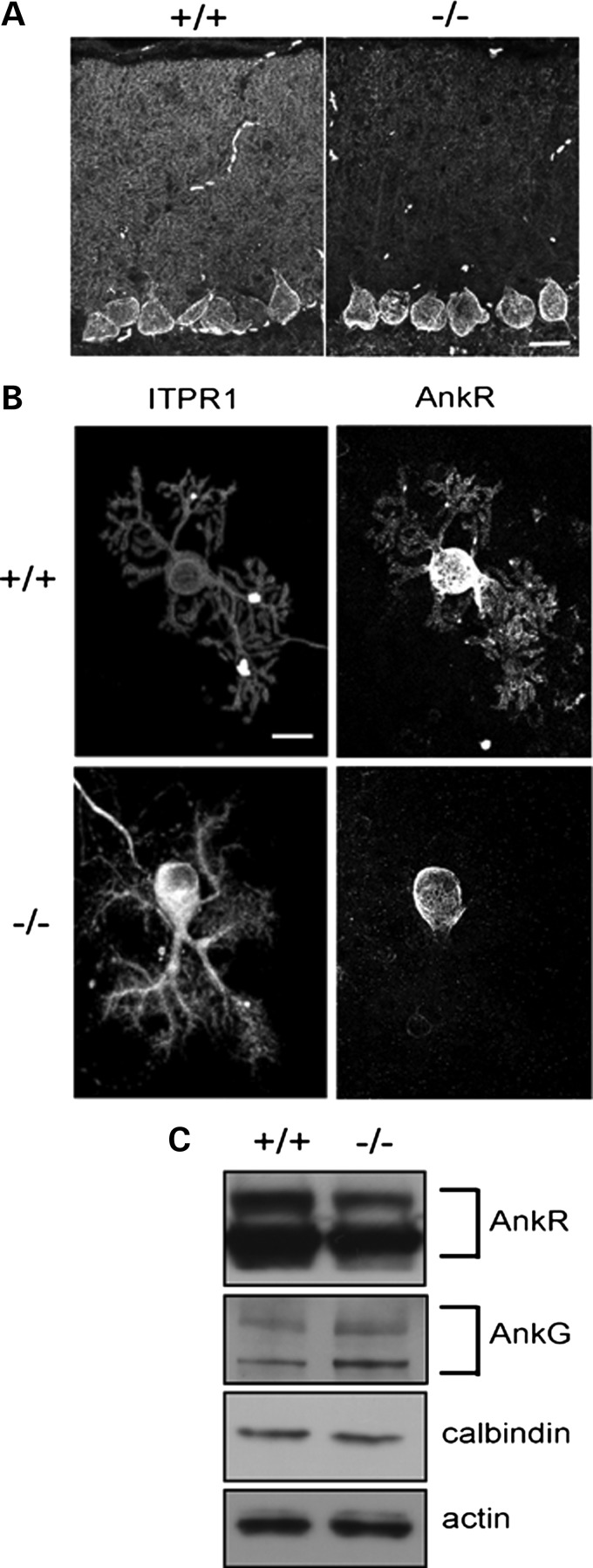
Loss of ankyrin R in β-III spectrin-deficient mice. (**A**) Sagittal cerebellar sections from 6-week-old WT (+/+) and β-III^−/−^ (−/−) mice immunostained for ankyrin R. (**B**) Dissociated Purkinje cell cultures from WT (+/+) and β-III^−/−^ (−/−) mice 14 DIV immunostained for ITPR1 and ankyrin R. (**C**) Immunoblot analysis of cerebellar homogenates from 6-week-old WT (+/+) and β-III^−/−^ (−/−) mice probed for ankyrin R, ankyrin G, calbindin and actin. All images are representative of at least three independent experiments. Scale bar, 20 μm.

We next looked to see whether a loss of β-III spectrin affected the cellular distribution or expression levels of ankyrin R in Purkinje cells. Immunofluorescence microscopy of cerebellar sections from 6-week-old mice lacking β-III spectrin (β-III^−/−^) showed a reduction in ankyrin R immunoreactivity throughout the cerebellar molecular layer compared with wild-type mice (Fig. 2A). However, there was no obvious loss within Purkinje cell bodies. This may be due to the fact that another β spectrin isoform (β-IIΣ2) is expressed in Purkinje cell bodies but not in dendrites (23). Immunostaining of dissociated Purkinje cells maintained *in vitro* for 14 days (14 DIV) (Fig. 2B) further revealed that the loss of ankyrin R in the absence of β-III spectrin was throughout the Purkinje cell dendritic tree. Under these conditions, cells lacking β-III spectrin (β-III^−/−^) have dendritic processes that are clearly delineated by ITPR1 staining. Ankyrin R is clearly present throughout the wild-type dendrites but undetectable in the absence of β-III spectrin (−/−; Fig. 2B). Finally, immunoblot analysis showed a significant reduction in ankyrin R protein in the cerebellum of β-III^−/−^ mice compared with littermate controls (Fig. 2C; 64 ± 9% of WT, *P* = 0.026, *n* = 4). There was no significant change in ankyrin G levels, but there was a trend for elevated levels (152 ± 22% of WT, *P* = 0.14, *n* = 4). Ankyrin R is therefore found throughout the dendritic arborization of Purkinje cells and β-III spectrin is required to maintain this localization.

### β-III spectrin stabilizes ankyrin R

**Figure 3.**
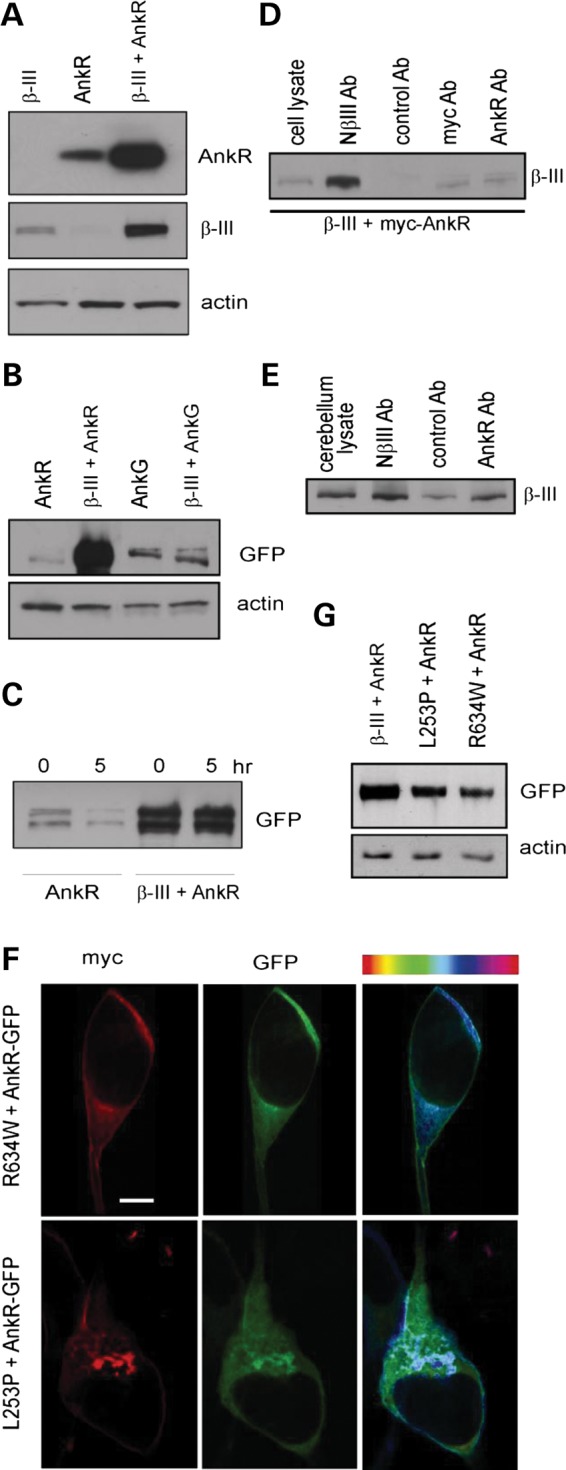
Ankyrin R and β-III spectrin interact and stabilize one another. (**A**) Immunoblot analysis of HEK 293T cell homogenates expressing β-III spectrin, myc-tagged ankyrin R or both proteins. Immunoblots probed with anti-AnkR, -β-III spectrin and -actin antibodies (latter for internal protein loading control). (**B**) Cell homogenates from cells transfected with either AnkG-GFP or AnkR-GFP and co-transfected with β-III spectrin probed with anti-GFP, and -actin antibody for internal protein loading control. (**C**) Cells transfected with AnkR-GFP alone or with β-III spectrin and incubated with cycloheximide 48 h after transfection. Immunoblot analysis of cell homogenates before and 5 h after addition of cycloheximide probed with anti-GFP antibody. (**D**) Immunoblot analysis of proteins eluted following immunoprecipitations from HEK 293T cells coexpressing untagged β-III spectrin and myc-tagged ankyrin R probed with anti-β-III spectrin antibody. (**E**) Immunoprecipitations from cerebellar homogenate probed with anti-β-III spectrin antibody. (**F**) Representative confocal images of cells cotransfected with SCA5-associated β-III spectrin mutations L253P or R634W and AnkR-GFP, immunostained using anti-c-myc antibody (red) and residual map showing degree of colocalization. (**G**) Immunoblot analysis of HEK 293T cell homogenates expressing AnkR-GFP with either WT, L253P or R634W β-III spectrin probed with anti-GFP antibody and anti-actin for protein loading control. All images are representative of at least three independent experiments. Scale bar, 10 μm.

Next we carried out a number of *in vitro* experiments to investigate the interaction and stability of the two proteins. HEK 293 T cells were transfected with constructs encoding wild-type β-III spectrin (β-III) and/or myc-tagged AnkR. Immunoblot analysis of cell homogenates, harvested 48 h after transfection, revealed that coexpression of the two proteins resulted in higher levels of each protein being present compared to when each protein was expressed individually (Fig. 3A). This effect was much greater for ankyrin R than for β-III spectrin (379 ± 83% of AnkR levels when expressed alone, *P* = 0.029, *n* = 5; 164 ± 11% of individual β-III spectrin expression level, *P* = 0.028, *n* = 5). However, no significant increase from individual expression levels was observed for anykrin G-GFP (93 ± 2% of AnkG alone, *P* = 0.18, Fig. 3B). To directly examine protein stability, cells were treated 48 h after transfection with cycloheximide, an inhibitor of protein synthesis. In the absence of β-III spectrin, the levels of AnkR-GFP were significantly reduced after 5 h of cycloheximide treatment (38 ± 3.5% of original level, *P* = 0.003, *n* = 3), whereas the coexpression of β-III spectrin resulted in no loss of AnkR-GFP (Fig. 3C), thus demonstrating β-III spectrin can stabilize ankyrin R at the plasma membrane.

Co-immunoprecipitation assays confirmed that the two proteins are tightly associated (Fig. 3D and E). Antibodies against myc-tagged AnkR (anti-c-myc and anti-AnkR) but not control antibodies were able to pull down β-III spectrin protein from HEK 293T homogenates (Fig. 3D) and similarly β-III spectrin was co-immunoprecipitated from cerebellar homogeneates by anti-AnkR antibodies to a greater extent than with control antibodies (Fig. 3E). Antibody against N-terminus β-III spectrin was used as a positive control for β-III spectrin immunoprecipitation (Fig. 3D and E).

We next examined the influence of β-III spectrin mutations associated with SCA5 on the localization of ankyrin R. Two missense mutations were investigated, L253P located within the actin binding domain and R634W found within the third spectrin repeat of β-III spectrin. AnkR-GFP was coexpressed in HEK 293T cells with myc-tagged forms of each mutant (Fig. 3F). Compared with wild-type β-III spectrin (Fig. 1C), significantly less AnkR-GFP was localized at the plasma membrane when R634W was coexpressed (41 ± 14% less than WT, *P* = 0.002, *n* = 15). In the case of L253P, the majority of transfected cells possessed intracellular accumulations of mutant β-III spectrin and AnkR-GFP with little of either protein at the membrane. Finally, immunoblot analysis revealed that coexpression of L253P and R634W with AnkR-GFP led to lower ankyrin R protein levels when compared with WT β-III spectrin (55 ± 7 ;58 ± 8% of WT levels, *P* < 0.01, *n* = 4, Fig. 3G).

### Expression of β-III spectrin and ankyrin R enhances sodium currents

**Figure 4.**
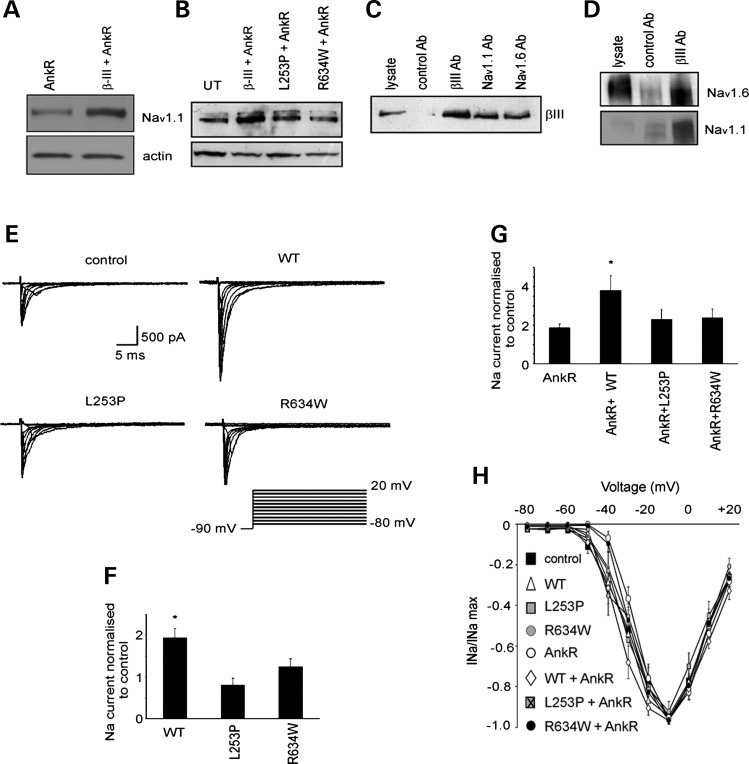
Sodium channel levels and activity enhanced by β-III spectrin/ankyrin R complex. (**A** and **B**) Immunoblot analysis of HEK 293T cell homogenates expressing ankyrin R alone or with either wild-type (WT) or SCA5-associated mutant forms β-III spectrin (L253P, R634W) probed with anti-Na_v_1.1 and -actin antibodies (latter for internal protein loading control). (**C** and **D**) Immunoprecipitations from cerebellar homogenate probed with anti-β-III spectrin (C), anti-Na_v_1.1 and - Na_v_1.6 antibodies (D). (**E**) Sodium current traces from representative cells evoked with a series of 50 ms depolarizations from a holding potential of −90 mV to potentials ranging from −80 to + 20 mV in 10 mV increments (stimulus protocol bottom right). (**F**) Sodium current peak at −10 mV normalized to control cells cultured at same time (either transfected with fluorophore only or untransfected). (**G**) Normalized sodium current for cells transfected with AnkR-GFP alone or with WT or mutant forms β-III spectrin. (**H**) Current–voltage relationships for all conditions with current amplitude normalized to peak value. All data are means ± SEM (*n* = 11–34; *P < 0.05).

Previously, we reported reduced sodium currents in Purkinje cells from β-III spectrin knockout (β-III^−/−^) animals (15). Since β-IV spectrin and ankyrin G are instrumental in clustering voltage-gated sodium channels along axons (24,25), we investigated whether expression of β-III spectrin and ankyrin R affected sodium channel expression. Na_v_1.1 and Na_v_1.6 are the predominant sodium channels in the soma and dendrites of Purkinje cells (26). We could only detect endogenous Na_v_1.1 in HEK 293T cells (data not shown) and so concentrated our analysis on this channel. We examined the effect coexpression of β-III spectrin and ankyrin R had on Na_v_1.1 levels in HEK 293T cells. Immunoblot analysis showed that Na_v_1.1 levels were higher when β-III spectrin and ankyrin R were coexpressed (190 ± 25% of ankyrin R levels, *P* = 0.035, *n* = 4, Fig. 4A), demonstrating a role for a β-III spectrin/AnkR complex in stabilizing Na_v_1.1 expression. In contrast, levels of Na_v_1.1 were lower when SCA5-associated mutants, L253P and R634W, were coexpressed with ankyrin R (64 ± 1; 55 ± 7% of WT levels, *P* < 0.05, *n* = 3, Fig. 4B). To test if the interactions detected in HEK 293T cells reflected endogenous complex formation, we examined cerebellar homogenates by co-immunoprecipitation. Antibodies against Na_v_1.1 and Na_v_1.6 efficiently co-purified β-III spectrin (Fig. 4C). Similarly, antibodies against β-III spectrin co-purified Na_v_1.1 and Na_v_1.6. (Fig. 4D).

We next tested if the interactions detected by immunoprecipitation had functional consequences on neuronal sodium currents. Purkinje cells are challenging to reliably transfect. Therefore, to examine the effect of β-III spectrin/ankyrin R interactions in a neuronal context, we measured sodium currents in dissociated hippocampal pyramidal neurons transfected with WT or mutant β-III spectrin and ankyrin R expression constructs. Electrophysiological recordings revealed an increase in peak sodium currents when WT β-III spectrin was expressed, whereas expression of either L253P or R634W had no effect on sodium currents with maximal current matching that of untransfected cells or cells expressing fluorophore only (Fig. 4E,F). Furthermore, when WT β-III spectrin was co-expressed with ankyrin R, a further increase in sodium currents was observed demonstrating an additive effect of a β-III spectrin/ankyrin R complex on sodium channel activity (Fig. 4G). However, there was no significant enhancement of ankyrin R effects when the SCA5-associated mutations, L253P or R634W, were co-expressed. There was no change to sodium channel kinetics when β-III spectrin, mutant forms of β-III spectrin or ankyrin R were expressed as voltage-dependent properties were unchanged, shown by superimposed current–voltage relationships for all conditions (Fig. 4H).

## DISCUSSION

This study is the first to show a direct interaction between β-III spectrin and erythroid ankyrin in the cerebellum and to demonstrate a critical role for β-III spectrin in maintaining ankyrin R throughout the Purkinje cell dendritic tree. Furthermore, our previous work revealed reduced sodium currents in acutely dissociated Purkinje cells from β-III^−/−^ mice, suggesting that one physiological consequence of β-III spectrin mutations found in SCA5 and SPARCA1 individuals may be reduced Purkinje cell output due to smaller sodium currents (15). Here, we have been able to directly demonstrate that β-III spectrin and ankyrin R positively modulate neuronal sodium currents. Moreover, this is the first study to show that two mutations in β-III spectrin associated with SCA5 negatively affect the ability of the protein to localize ankyrin R to the membrane, and result in reduced sodium currents.

Spectrins contain a central repeat region consisting of 17 spectrin repeats. It has previously been shown that a particular orientation of spectrin repeats 14 and 15 is critical for stable complex formation with ankyrin and that mutations in β-I spectrin affect the folding stability of spectrin (27). Therefore, although all the disease-causing mutations identified to date in β-III spectrin lie out with the ankyrin binding domain, a phenomenon in common between them may be alteration of the overall tetrameric conformation of spectrin such that the stability of interaction with ankyrin is no longer optimal. A similar situation has been observed for the Purkinje cell glutamate transporter EAAT4, where an in-frame deletion in the third spectrin repeat identified from the Lincoln SCA5 pedigree disrupted stabilization of EAAT4 at the cell surface compared with wild-type β-III spectrin (17), even though EAAT4 interacts with the unaffected C-terminus of β-III spectrin (28).

The missense mutation R634W used in this study was found in a French pedigree in combination with a five amino acids in frame deletion (17). Therefore, it was unclear whether the substitution of arginine for tryptophan at position 634, the in-frame deletion (Leu-Ala-Ala-Ala-Arg) or a combination of both genetic defects is pathogenic. Here, we provide strong evidence that the R634W itself has a significant negative effect on β-III spectrin function, but we cannot rule out the possibility that the in-frame deletion might exacerbate the observed defects. However, there is no evidence for any correlation between clinical presentation and genetic mutation, suggesting that the different mutations actually impact β-III spectrin function to similar degrees.

The importance of ankyrin R for normal red blood cell biology is well known, but less is known regarding its roles within the nervous system. Here, we show for the first time that ankyrin R, in association with β-III spectrin, has a critical role in modulating neuronal sodium channel activity and hence neuron excitability. This provides a physiological explanation for why motor defects, including loss of balance and broad-based gait, are observed in individuals with hereditary spherocytosis deficient in ankyrin R (29,30). The inability to maintain ankyrin R throughout the plasma membrane of Purkinje cell dendrites may also be a critical factor in the development of abnormal dendrite morphology observed in the absence of β-III spectrin (16). As far as we are aware, the three-dimensional structure of Purkinje cells from the *nb/nb* mouse has not been examined, but it would be interesting to see whether lack of ankyrin R in these mice leads to thinner and disorientated dendrites as seen in β-III^−/−^ mice. What is noteworthy is that in both mice, the genetic loss of either β-III spectrin or ankyrin R is absolute throughout development and yet Purkinje cell loss is only observed about 6 months of age. Therefore, the late onset neurological symptoms and neurodegeneration appear to be a consequence of accumulating neuronal dysfunction due to protein deficiency from the outset. One possibility is that a reduced sodium channel density in Purkinje cell dendrites due to disruption of a β-III spectrin/ankyrin R complex, and in conjunction with an altered dendritic geometry, affects propagation of dendritic action potentials and thus integration of synaptic inputs (31). Changes to the dendritic expression of sodium channels may also affect cell–cell adhesion interactions as the auxiliary β subunits are thought to regulate cell adhesion and migration (32).

The fact ankyrin G defects have also been associated with autism susceptibility (33), bipolar disorder and schizophrenia (34) implicates other members of the ankyrin family in neurological disorders. How these mutations cause the phenotype is not known, but it was hypothesized that ankyrin G mutations may lead to the abnormal development of neurons and affect neuronal excitability through altered ion channel function (33). Here, we actually show that mutations in β-III spectrin associated with SCA5 affect ankyrin R levels and sodium channel activity pointing toward common mechanisms in a wide spectrum of neurological disorders.

In conclusion, we have shown that β-III spectrin interacts with and maintains ankyrin R at the plasma membrane throughout the Purkinje cell dendritic tree and that this interaction is critical for normal sodium channel activity in neurons. Furthermore, impairment of this interaction appears to be a consequence of mutations found in β-III spectrin associated with human ataxia. Identifying ways to enhance levels of ankyrin R and associated proteins at the cell membrane may, therefore, be one therapeutic strategy worth investigating for this group of debilitating disorders.

## MATERIALS AND METHODS

### Plasmids

Plasmids encoding ankyrin-G-GFP and myc-tagged ankyrin-R were a kind gift from Vann Bennett. Full-length Ankyrin-R-GFP and spectrin binding domain Ankyrin-R-GFP plasmids were constructed using human cDNA clone as template and PCR primers that introduced *Eco*RI restriction sites. PCR products were cloned into *Eco*RI site of pEGFP-N1 in frame with GFP. Other plasmids were myc-tagged WT, L253P and R634W rat β-III spectrin in pRK5 and untagged β-III spectrin in pCDNA3.1. Missense mutations were introduced using the QuickChange site-directed mutagenesis kit (Stratagene) according to the manufacturer's instructions using pRK5-myc-tagged β-III spectrin as template.

### Antibodies

Primary antibodies used were rabbit anti-GFP (Invitrogen), -Nav1.1 (Millipore), -ITPR1 (Millipore), -ankyrin-G (Santa Cruz), - β III spectrin (kind gift of Jeffrey Rothstein), mouse anti-ankyrin-R (Santa Cruz), -actin (Sigma), -c-myc (Calbiochem), -calbindin (Swant) and goat anti-β III spectrin (Santa Cruz). Secondary antibodies were fluorescein isothiocyanate-conjugated goat anti-rabbit IgG (Cappel), cyanine 3 (Cy3)-conjugated goat anti-mouse IgG, Cy3-conjugated donkey anti-goat IgG (Jackson Laboratories), HRP-conjugated donkey anti-rabbit IgG, HRP-conjugated sheep anti-mouse IgG, HRP-conjugated donkey anti-goat IgG (Amersham Pharmacia).

### Immunoprecipitations

Adult mouse brains were rapidly dissected and homogenized in buffer (20 mm HEPES pH 7.4, 2 mm EDTA, protease inhibitors and PMSF). Triton X-100 was added (1% final v/v) and homogenate solubilized for 30 min at 37°C. Transfected HEK 293T cells were plated onto 10-mm dishes and solubilized 48 h post-transfection. For both homogenates and cells, cellular debris was removed by centrifugation at 15 000*g* for 20 min, the supernatant removed and 2 μg of antibody added followed by rotation overnight at 4°C. Twenty microlitres of protein G agarose bead suspension (Sigma) was then added and mixed for 2 h at 4°C. The beads were washed four times and bound proteins released by heating at 60°C for 10 min before being separated by SDS–PAGE and detected by immunoblotting as previously described (15) or using ECL Prime western blotting detection reagent (GE Healthcare).

### Protein stability

HEK 293T cells were grown as previously described (35), transfected at 60–70% confluence in six-well plate and 48 h after transfection cycloheximide was added at a concentration of 10 μg/ml. Cells were harvested at 0 and 5 h after cycloheximide treatment and processed for immunoblot analysis by resuspending in homogenization buffer.

### Immunofluorescence microscopy

For paraffin sections, brains were removed and immersion-fixed with 4% paraformaldehyde in 0.1 m sodium phosphate buffer, pH 7.4 overnight at 4°C prior to embedding in paraffin. Sections (10 μm) were cut and mounted onto poly-L-lysine coated slides. Dissociated Purkinje cell cultures were prepared as previously described (36) except that cerebella were dissected at P0, digested in papain (Worthington) and dissociated cells were plated on poly-L-lysine coated cover slips. HEK 293T cells were plated onto cover slips coated with poly-l-lysine in 35 mm dishes and transfected with 250 ng of each DNA construct using Fugene HD reagent (Roche, Indianapolis, IN, USA) in accordance with the manufacturer's instructions. All immunostaining was carried out as described previously (15) and images were captured with a Zeiss inverted LSM510 confocal laser scanning microscope. All acquisition settings were kept constant between samples, except for colocalization analysis and colours applied using Image J. For colocalization analysis, the gain was adjusted for each cell and degree of colocalization calculated using Pearson's correlation coefficient. Residual maps were generated to visually represent varying degrees of colocalization and degree of colocalization at membrane quantified by removing all colours except cyan (high colocalization).

### Hippocampal cultures and electrophysiology

Primary rat hippocampal neuronal cultures were prepared from embryonic day 18 Sprague–Dawley embryos as previously described (37) and transfection of cells performed using the Amaxa Rat Neuron Nucleofector Kit (Lonza) according to the manufacturer's instructions. Control cells transfected with fluorophore construct only were plated on the same cover slips as cells transfected with constructs expressing β-III spectrin and/or ankyrin-R, spiked with a different fluorophore to that of control cells. From a single cover slip recordings could then be made from neurons untransfected, transfected with a fluorophore only or transfected with expression constructs and fluorophore. No difference was observed between untransfected and fluorophore only cells and so data from those cells were pooled. The control extracellular recording solution contained (in mm) 130 NaCl; 20 TEA-Cl; 3 KCl; 1 MgCl_2_; 1 CaCl_2_: 0.3 CdCl_2_; 10 HEPES and 10 glucose, buffered to pH 7.4 with NaOH. Recordings were made at room temperature on cultures 7–8 DIV with borosilicate pipettes (3–5 MΩ) containing (in mm): 140 CsF; 1 EGTA; 10 NaCl and 10 HEPES adjusted to pH 7.4 with CsOH. To isolate the TTX-sensitive Na^+^ current, voltage protocols were repeated in the presence of 300 nm TTX and subtracted from the control recordings. Data were acquired using pClamp 10 (Axon Instruments, Foster City, CA, USA) and analysed using IGOR Pro (Wavemetrics, Lake Oswego, OR, USA).

### Statistical analysis

Statistical analysis was performed using Student's *t*-test, two samples assuming unequal variance or one-way ANOVA.

## FUNDING

This work was supported by grant from The Wellcome Trust (093077). Funding to pay the Open Access publication charges for this article was provided by the Wellcome Trust.
